# Living a burdensome and demanding life: A qualitative systematic review of the patients experiences of peripheral arterial disease

**DOI:** 10.1371/journal.pone.0207456

**Published:** 2018-11-15

**Authors:** Ukachukwu Okoroafor Abaraogu, Elochukwu Fortune Ezenwankwo, Philippa Margaret Dall, Chris Andrew Seenan

**Affiliations:** 1 University of Nigeria Department of Medical Rehabilitation, Enugu, Nigeria; 2 Glasgow Caledonian University School of Health and Life Sciences, Glasgow, United Kingdom; Keele University School of Medicine, UNITED KINGDOM

## Abstract

**Background:**

Peripheral arterial disease (PAD) has a significant negative impact on the quality of life of individuals. Understanding the experiences of people living with PAD will be useful in developing comprehensive patient-centred secondary prevention therapies for this population.

**Aim:**

The aim of this study is to identify first-hand accounts of patients’ experiences of living with PAD.

**Methods:**

Six databases (CINALH, PsyclNFO, MEDLINE, AMED, EMBASE, Social citation index/Science citation index via Web of Science (WOS)) and reference lists of identified studies were searched until September 2017 (updated February 2018). Qualitative studies reporting patients’ account of living with PAD were eligible for inclusion. A framework thematic synthesis was implemented.

**Results:**

Fourteen studies with 360 participants were included. Pain and walking limitation were recurrent among the varied symptom descriptions. Patients’ ignorance and trivialisation of symptoms contributed to delays in diagnosis. Inadequate engagement in disease understanding and treatment decisions meant patients had poor attitudes towards walking treatments and unrealistic expectations about surgery. Depending on symptom progression, patients battle with walking impairment, powerlessness, and loss of independence which were a source of burden to them. Lack of disease understanding is central through patients’ journey with PAD and, although they subsequently began adaptation to long term living with PAD, many worried about their future.

**Conclusions:**

Disease understanding is vital across the illness trajectory in patients with PAD. Although certain experiences are common throughout patient journey, some might be unique to a particular stage (e.g. unrealistic expectation about surgery, or rationale of walking in spite of pain in a supervised exercise program). Given that PAD is an overarching construct ranging from the mildest form of intermittent claudication to severe critical limb ischemia with ulceration and gangrene, consideration of important patient constructs specific to each stage of the disease may enhance treatment success. Systematic review registration CRD42017070417.

## Introduction

Peripheral arterial disease (PAD) has a pronounced negative effect on individuals quality of life [1–3]. Although with a fairly common pathophysiology, risk factors, and management pathway, symptom presentation in greater majority of patients with PAD is often atypical and difficult to predict [4]. One important aspect of PAD is the fact that it is mainly a symptom of a general atherosclerotic disease often presenting with a high comorbidity rate making secondary prevention, in a long term treatment approach, an important part of their management. Also, the general atherosclerosis as well as concomitant high comorbidity rates among this group of patients has implications for their health-related quality of life and disease experience.

There is a growing interest in patient-centred care for the management of PAD [5]. However, the manner in which a patient interacts with the management of their condition or the symptom presentations throughout the care pathway may be influenced by their understanding of the disease [6].Therefore, qualitative studies exploring patients experiences of living with PAD may enable a better understanding of how patients adapt and cope with the disease, enabling better conceptualisation of patients’ decisions regarding available or future management.

Understanding the experiences of people living with PAD via a qualitative synthesis will be useful in developing comprehensive patient-centred secondary prevention therapies for this population. In addition, it may allow tailoring of the future research agenda to investigate constructs important to patients and the management of PAD. The aim of this study is to provide an overview of patients’ experiences of living with PAD.

## Methods

**Fig 1:**
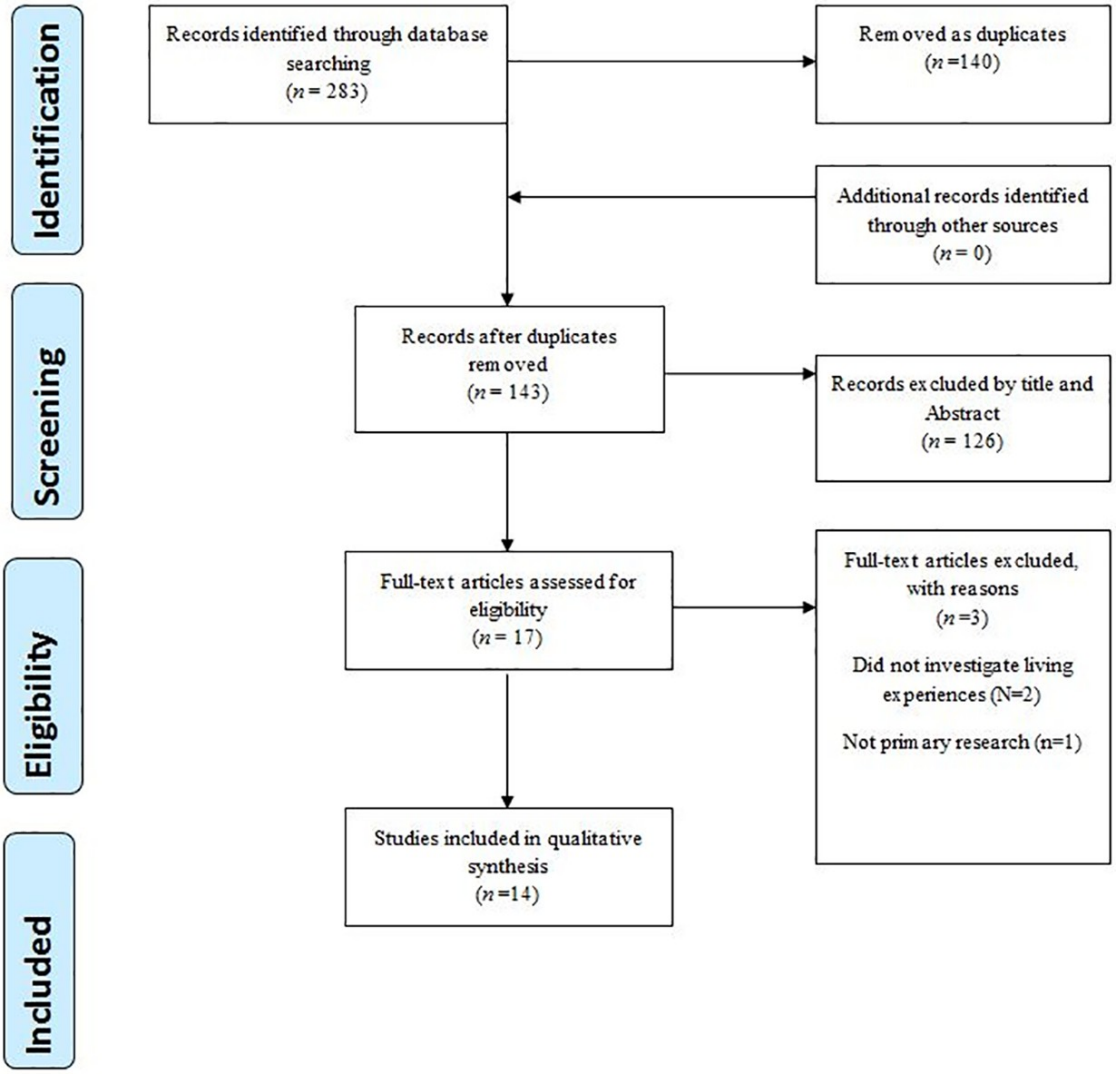
PRISMA flow diagram for patients experiences of living with peripheral arterial disease.

Preferred Reporting Items for Systematic Reviews and Meta-Analyses (PRISMA) (where applicable) and the four-phase item flow diagram guidelines were followed[7] (See Fig 1). The review protocol was registered in the PROSPERO systematic review register (CRD42017070417). Fig 1 summarizes the flow of studies from identification to inclusion. The PRISMA checklist for this review is shown in the supporting information (S1 Table).

### Eligibility criteria

#### Type of participants

Studies that reported on adults (≥18 years) with PAD were included. Studies were included whether there was a diagnosis of PAD, or PAD was stated or confirmed as the underlying cause.

#### Types of studies

Studies on self-reported accounts of patients’ experience of being diagnosed and treated for PAD were included if published in English and reported primary data. Also mixed-methods studies were included if they reported the qualitative results separately. Survey only or hypothetical data studies were excluded. Studies published only as abstracts were excluded.

### Information sources, search strategy, study records, and data management

#### Identification of primary research studies

The search aimed to find only peer review published studies. A systematic search was conducted until September 2017 (Updated in February 2018) in six databases (CINALH via EBSCO, PsyclNFO via Ovid, MEDLINE via Ovid, AMED via Ovid, EMBASE via Ovid, Social citation index/Science citation index via Web of Science (WOS)) and directory of Open Access repository websites by the first author (UA). The SPIDER tool [8] was used to organise the research questions and the search blocks. The search strategies were adapted to each database specific search syntax, filters, limiters and Boolean operators. Generally the following key words were used: Peripheral arterial disease OR Peripheral vascular disease OR Peripheral Occlusive disease OR Intermittent claudication OR Claudication pain AND Quality of life OR Life experience OR Lived experience OR Patient reported experience OR Patient experience OR Experience of living OR Experiences OR Illness beliefs AND qualitative OR mixed-methods. Reference lists of identified studies were also searched. A sample search strategy is presented in the supporting information (S1 Fig). The identified studies were imported to Refworks and duplicates removed. Studies were then exported to excel. Titles, abstracts, and the full text of selected studies were independently screened by two authors (UA, EE) using the previously defined eligibility criteria. Differences of opinion regarding inclusion or exclusion were resolved by discussion and reaching consensus between the two authors (UA, EE), or in consultation with a third author (CS) when warranted.

### Data extraction processes

Study characteristics were recorded in a data extraction form specifically developed and piloted for this review (Table 1). Data elements included authors’ details, phenomenon of interest, participant characteristics, study design and qualitative findings and themes. All studies had information sufficient to enable data analysis; hence no further contact to study authors was warranted. Specifically, both patients’ quotes (primary data) and emerging themes (secondary data) from included studies were extracted to aid the framework synthesis.

**Table 1:** Table of data extraction and characteristics of included studies.

Study, date, country	Study design, sampling, data collection and analysis methods	Participants, Age, Gender	Phenomenon of interest	Setting,	Geographical, Cultural	Key outcomes, Findings, authors conclusion
Galea et al[10] United Kingdom	Qualitative semi-structured in-depth individual face-to-face interviews: CSM+TPB. Framework analysis	Participants: n = 19 *Gender*:13M *Age*: mean 66 (range 44–79)y *Disease duration*: ≥2y = 10(53%); <2y = 9(47%) *Past treatments*: Revascularization = 5 (26%); SEP = 8(42%)	Experiences of and belief about illness and walking with IC	Patient home/ University facility	White: 12(63%) Ethnic minorities: 7(57%)	Walking is an overlooked self-management treatment Patient desired a tailored walking guidance Patient had varied outcome expectation from walking Patients lack specific instruction about walking (e.g. walking through pain) Patients had uncertainty about the appropriate walking intensity barriers
Harwood et al[11] United Kingdom	Qualitative semi-structured interview Thematic analysis	Participants: n = 25 Gender: 14(56%)M; Age: mean = 71, range = 44–79 Stable IC Patients who declined, withdrew from, or completed supervised exercise program.	Patients experience of diagnosis and treatment with a supervised exercise program	Patient home/ Hospital facility	Not documented	Theme and Subthemes *Intermittent claudication* -Understanding disease and risk factors -Effect on lifestyle and daily activities -Consultants provide the best medical therapy and advice *Perception of exercise* -preview experience with exercise -Present activity level -Exercise benefits *Experience of exercise program* -Class experience -Motivation for adherence to exercise program -Program repetition -Barrier to exercise class -Enjoyment to exercise
Hallin et al[12] Sweden	Mixed study (cross-sectional study); Sampling: Not clear; Semi-structured interview; Content analysis	Participants: n = 80 Age = 56-88y Gender = 47(59%)M Type of disease CAS; AAA; IC; CLI (n = 20 each) Treatment planned or already performed Conservative/ follow up(n = 17) Planned op or PTA (n = 21 Operated or PTA elective/Acute (n = 41) Not operated (n = 1)	Patients concerns and subjective effect of PAD on quality of life and life satisfaction	Clinic facility/Patient home	Swedish	Physical functioning Social functioning Sexual functioning Anxiety/concern Intellectual capacity Family concern Pain Factors related to surgery There is a need to incorporate disease specific questions to generic quality of life instruments and measurements of life satisfaction to fully understand how PAD affects individuals
Schorr et al[13] USA	Qualitative research; Purposive sampling; Semi-structured face-to-face interview and PAD symptom question; Content analysis	Participants: n = 38 Stage of disease: Not specified (Generic) Mean age: 67.58y; Race: Caucasian 89.95% Gender: 33M; 5F Age: 57.9% Older adults(≥65y): Education: 85% attending some collage or graduate school Employment: 63. 2% retired Marital status: 60.5% Married/living with partner	Symptoms experience of patients diagnosed with PAD	Hospital facility	Predominantly Caucasians; African Americans; Native Americans	Six themes: Symptom descriptors (Claudication and atypical); maintaining equilibrium; Temporal fluctuation; the role of exercise; Perceived impact on quality of life; Disease presence and treatment.
Johnstone[14] UK	Ethnography; Convenient sampling; One-on-one interview of couple; Framework analysis	Participants: n = 14 7 couples	Life experiences of patients with PAD and their carers	Patients home	Not documented	2 concepts: Acceptance and Adaptation 4 themes: The experience of pain; Consequence of impaired mobility; Loss of independence; Disease progress; Professional support.
Gibson and Kenrick[15] UK	Phenomenology; Convenient sampling; One-on-one interview & Grounded theory method	Participants: n = 9 Patients with PAD 3–18 post constructive surgery Gender: M = 6; F = 3 Age: (62-75years).	Patients experience of living with PAD; and how treatment affect individuals coping strategies	Patients home	English	4 themes: Pain; Someone else’s problem (patient hood, expectation, playing by the rules), Shrinking of horizons; Control, choice and changing outlook. Findings: Patients with PAD appear to experience powerlessness in relation to the direct effect of their condition as well as its treatment modalities. Conclusion: The acute style of management often led to unrealistic expectations from patients, giving rise to experience of powerlessness.
Egberg et al.[16] Sweden	Qualitative descriptive design, Purposive sampling; One-on-One interview Thematic analysis;	Participants: n = 15 Patients with PAD/IC awaiting visit or revisits to the vascular surgery Gender: M = 8; F = 7 Age: (64-81years)	Patients experiences of living with PAD/IC	Patients home, Researchers workplace, hospital	Swedish	6 themes: Experiencing discomfort in the legs; Moving around in a new way; Feeling inconvenient when forced to stop; Missing previous life; Incorporating IC in daily life; To lead a strenuous life. Findings: Living with IC has a major impact on daily life and demands adjusting to restricted life. Conclusion: Experience of living with IC depends on how active a patient is or wants to be, and understanding this experience is an important to complement treatment planning.
Gorely et al.[17] UK	Qualitative design; Purposive sampling; Focus group Thematic analysis	Participants: n = 24 Patients with PAD/IC recruited via screening vascular clinic letter Gender: M = 17; F = 7 Mean age: (71±8years)	Experiences, knowledge and beliefs of patients with IC.	Not documented	White British	2 themes: Uncertainly; Lack of support/empathy Findings: PAD/IC has significant impact on patients, including loss of activity and enjoyment. In addition, patients feel uncertain about the disease, and that medical professionals do not show empathy. Conclusions: Addressing the knowledge gaps and uncertainty around IC disease processes and walking will be key to providing behavior change in people with IC.
Treat-Jacobson et al.[18] USA	Grounded theory, Purposive sampling, Open-ended interview &Thematic analysis	Participants: n = 38 Patients of wide range of PAD severity Gender: M = 24; F = 14. Age: 44–83 (mean: 65±10.4) years	Effect of PAD on health related quality of life from the patients perspective	Facility outside hospital	American	7 themes: Delay in diagnosis and frustration in managing disease; Pain; Limitation in physical functioning; Limitation in social and role functioning, Compromise of self; Uncertainty and fear; Adaptation to the effect of the disease and demonstration of resiliency. Findings: In addition to physical limitation, living with PAD entails social and role limitations. The wider effects of PAD affect broad aspects of patients live. Conclusion: Patients with PAD experience significant physical, psychological, and emotional disabilities that are not easily assessed by available questionnaires.
Wann-Hassan & Wennick[19]	Inductive qualitative design; Purposive sampling; Focus group & Content analysis	Participants: n = 21 Patients with PAD who had undergone a vascular intervention during the preceding 6 months Gender: M = 9; F = 12 Age: (50-81years)	Patients experiences of PAD and how they communicate their knowledge about their illness and treatment	Hospital conference room	Swedish	4 categories: Describing the known and unknown; Conflicting feeling towards smoking; Feeling relieved yet uncertain; Consulting various sources of information. Findings: Patients with PAD navigate through uncertainty, beliefs and facts about their illness and treatment with palpable need of further knowledge of their condition and strategies for risk factor prevention. Conclusions: Following endovascular treatment, the amount of time spent with patients with PAD requires guidance in clinical practice to meet individuals’ need.
Wann-Hassan et al.[20] Sweden	Inductive qualitative design, Purposive sampling, One-on-One Semi-structured interview Content analysis	Participants: n = 24 PAD patients with IC awaiting intervention Gender: M = 12; F = 12 Age: (60-92years)	Patients experiences of living with PAD, and the influence on activity of daily living	Patients home	Swedish	3 themes: Being limited by the burden; Striving to relieve the burden; Accepting and adapting to the feeling of burden. Findings: Living with PAD and often entails major physical, social, emotional burden, leaving patients struggling for relief. Pain is a major feature. Conclusion: Structured education about PAD, including education about, and evaluation of pain pathology and management, exploring patient capacity is needed in managing people with PAD.
Wann-Hassan et al.[21] Sweden	Qualitative design, Convenience sampling, One-on-One interview Content analysis	Participants: n = 14 Patients with PAD 6 months, and 2½years after revascularization Gender: M = 9; F = 5 Age: (61-85years)	Long term experience of living with PAD and the recovery after revascularization.	Patients home	Swedish	3 themes: Becoming better but not cured; Recapturing control over life; Reappraising meaning in life Findings: The expectations of people with PAD about recovery and the benefits of revascularization were initially unrealistic, but over time following revascularization, they gradually become aware of the chronic disease nature of condition. Conclusion: The long-term experience of living with PAD means becoming aware of it chronic nature highlighting the importance of structured education as a complementary for patients with PAD.
Cunningham et al.[22] UK	Qualitative design, Convenience sampling, Semi-structured interview Thematic analysis	Participants: n = 20 Patients with PAD/IC and is between 6 months and 2years after revascularizations. Gender: M = 11; F = 9 Age: (mean 70.9±6.6)	Belief and walking behavior of patients with PAD/IC after vascular intervention	Not documented	Scottish	5 themes: Walking avoidance; Treatable condition; Causal belief; Perceived consequences; Surgeon patient communication Findings: People with PAD appear to have impaired and incoherent beliefs about PAD/IC, treatment, and health behavior, and therefore do not change PA behavior after diagnosis and treatment for IC. Conclusion: There is a gap between the beliefs of patients with PAD/IC about their condition and the recommendation to increase PA that may affect their PA behavior.
Lokin et al.[23] Netherlands	Qualitative design; Convenience stratified by gender; Semi-structured one-on-one interview; Thematic analysis	Participants: n = 19 Patients with IC from iliac obstructions who have undergone either percutaneous angioplasty (PTA) or supervised exercise therapy. Gender: M = 10; F = 9 Age: (Mean: 60.8; Range: 51-72y)	The disease understanding of patients with IC regarding etiology factor and systemic nature of their disease	Not documented	Dutch	2 themes: Etiology; Perception of the systematic nature of IC. Findings: Patients living with IC have poor disease understanding particularly the etiology and systemic nature of their problem and this may hinder lifestyle medication and shared decision making. Conclusion: A change of counselling strategies is needed to better effect lifestyle modification and decrease cardiovascular morbidity and mortality in patient with in patients with PAD/IC. This change may warrant additional role for health care practitioners who have more extensive contact with the patients.

Key: CSM (common sense model), TPB (theory of planned behaviour)

### Critical appraisal

The Qualitative research Critical Appraisal Program (CASP) instrument was used to assess the methodological quality of each of the included studies [9]. CASP has been used in several qualitative systematic reviews and was deemed a suitable appraisal tool for the current review. It is a generic quality appraisal tool and it was anticipated that included studies would repor**t** diverse range of methodological underpinnings and research design.

### Data synthesis

**Fig 2:**
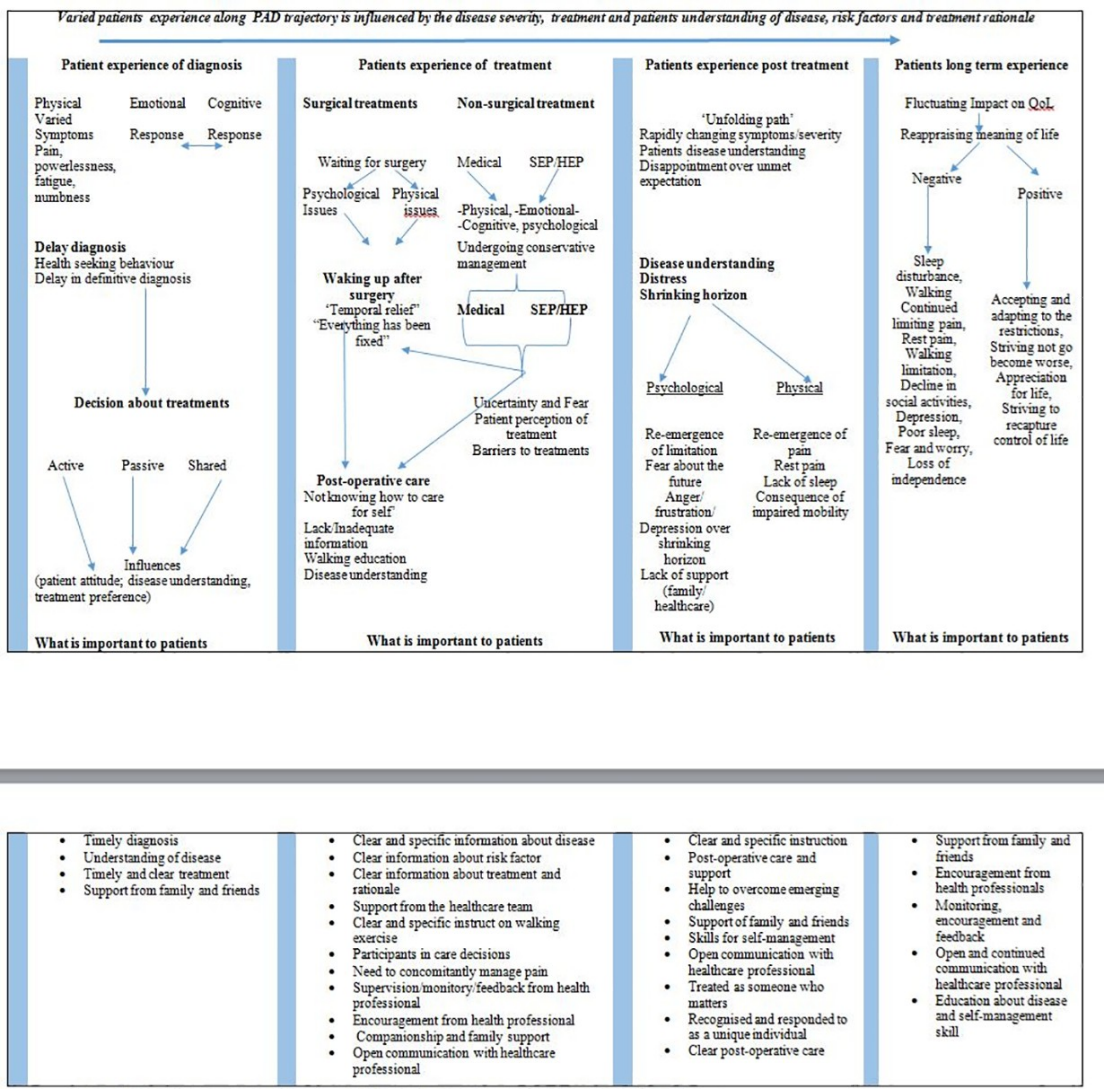
Framework synthesis of patients experiences of living with peripheral arterial disease.

A ‘best fit’ framework synthesis approach [24] was used to implement a preliminary deductive thematic analysis of the identified patients’ experiences of PAD. Given that PAD is a chronic disease, and the review aimed to report experience across the spectrum of PAD disease trajectory, theme allocation to the framework was undertaken with the perspective of PAD as a chronic disease and used emerging findings and themes in included studies. The synthesized framework is considered preliminary and will form the basis for further work on conceptualizing disease experience framework in this patient population. Allocation of themes were undertaken starting with the experience of diagnosis, experience of receiving acute care (interventions) treatments, experience of undergoing conservative treatments (e.g. supervised exercise program(SEP)), experience post-treatment, and long-term experience of living with PAD (the new normal). These themes informed the framework as they describe the patients’ pathway from diagnosis to adapting (or not) to living with PAD hence fit with the review aim. Understanding that different disease stages may present at the same treatment point, attempts, where possible, were made to present experience in relation to disease severity. Initial allocation of experiences to the framework as well as identifying emerging themes was initially undertaken by the first author (UOA) aided by discussions among the team. Agreement on a final themes and allocation was reached by discussion and consensus. Fig 2 provides an audit log to facilitate readers understanding of the synthesis and the preliminary framework.

## Results

Initial search results yielded a total of 283 articles, of which 140 were removed as duplicates. One hundred and twenty six papers were excluded after screening article titles and abstracts. Three papers were further excluded following full-text reading and eligibility assessment. Two of the studies[25,26] did not investigate patient reported constructs of living experiences of PAD/IC; and one[27] was not a primary research. Therefore, a total of fourteen studies were available for the qualitative synthesis. Fig 1 presents a flow chart of the search through to studies inclusion.

### Included studies and participants characteristics

Account of 360 patients of which 213(59.2%) were male and 133(36.9%) were female (gender missing in one study, n = 14), with age range from 44 to 92 years are reported. Most studies were from the United Kingdom (n = 6) or Sweden (n = 6). Two were conducted in the USA and one in the Netherlands (Table 1). Where reported, interview setting ranged from patients’ homes[10–12,14–16,20,21], researcher’s workplace[16], to facilities outside[18] or within the hospital[11–13,19] or university[10]. All participants in included studies were stated to have been diagnosed with PAD, but majority of the studies[12,14–16,18–22] did not report diagnostic criteria. Where reported the basis for confirming PAD ranged from ABI≤0.90, post-exercise drop in ABI, angiography, computed tomography or magnetic resonance imaging (MRI) scans.

Disease stages of participants was not reported in two studies[14,15] and varied within and across other studies which reported it. Five studies[12,18–21] included participants at various stages of the disease: intermittent claudication(IC), chronic limb ischaemic (CLI), and ischemic ulcer). The remaining studies[10,11,13,16,17,22,23] included only patients with symptoms of IC. There were similar variations within and across included studies about the type of treatment participants were undergoing. Four studies[12–14,18] described disease experience across the full spectrum of PAD. Where specified, majority of the studies (n = 6) [15,16,19–22] focused on patients disease experiences and understanding, and these constructs while awaiting or following surgical interventions. Three[10,11,17] investigated patients experience of disease and their perception of walking managements. One study[23] described the understanding in patients who have undergone either a SEP or percutaneous transluminal angioplasty(PTA). Where reported, there were wide variations in the duration since diagnosis or post-intervention for PAD, ranging between studies with participants at the different stages of the disease awaiting intervention[20] to a study with participants up to 2½ years post revascularization[22].

### Quality appraisal

The qualitative research CASP quality tool is marked out of a possible 10 ‘yes’ responses. All studies were included in the review irrespective of their quality rating, however, the rating was used as an indication of strength of evidence and to guide recommendation regarding required standard to inform future research on qualitative experience of patients with PAD. Seven studies[10,11,15,18–21] were awarded nine out of a possible 10 ‘yes’ responses; five studies[14,16,17,22] were awarded eight ‘yes’ responses. One study each was awarded 10[13], six[23], and five[12] ‘yes’ responses. The main source or potential source of bias in included studies was uncertainty about the role of the researcher, or any attempt to counter potential researcher bias on data collection (n = 12). There were also issues related to appropriateness of the research design to address the aims of the research (n = 2), the data collection in a way that addressed the research issue (n = 2), lack of ethical consideration (n = 2), issues related sufficient rigour in data analysis (n = 2). Table 2 presents the detail of the quality appraisal of included studies.

**Table 2:** Table of quality appraisal of the included studies using the CASP qualitative research checklist.

	Was there a clear statement of the aims of the research?	Is there a qualitative design appropriate?	Was the research design appropriate to address the aims of the research?	Was the recruitment strategy appropriate to the aim of the research?	Was the data collected in a way that addressed the research issue>	Has relationship with the researcher been adequately considered?	Have ethical issues been taken into consideration?	Was the data analysis sufficiently rigorous?	Is there a clear statement of findings?	How valuable is the research?
Galea et al[10]	Yes	Yes	Yes	Yes	Yes	Can’t tell	Yes	Yes	Yes	Yes
Harwood et al[11]	Yes	Yes	Yes	Yes	Yes	Can’t tell	Yes	Yes	Yes	Yes
Schorr et al.[13]	Yes	Yes	Yes	Yes	Yes	Yes	Yes	Yes	Yes	Yes
Johnstone[14]	Yes	Yes	Yes	Can’t tell	Yes	Can’t tell	Yes	Yes	Yes	Yes
Gibson & Kenrick[15]	Yes	Yes	Yes	No	Yes	Yes	Yes	Yes	Yes	Yes
Egberg et al.[16]	Yes	Yes	Yes	Yes	No	Can’t tell	Yes	Yes	Yes	Yes
Gorely et al.[17]	Yes	Yes	Yes	Yes	Yes	Can’t tell	Can’t tell	Yes	Yes	Yes
Hallin et al.[12]	Yes	No	Can’t tell	Yes	Can’t tell	No	Yes	Can’t tell	Yes	Yes
Treat-Jacobson et al.[18]	Yes	Yes	Yes	Yes	Yes	No	Yes	Yes	Yes	Yes
Wann-Hansson & Wennick[19]	Yes	Yes	Yes	Yes	Yes	No	Yes	Yes	Yes	Yes
Wann-Hansson et al.[20]	Yes	Yes	Yes	Yes	Yes	No	Yes	Yes	Yes	Yes
Wann-Hansson et al.[21]	Yes	Yes	Yes	Yes	Yes	No	Yes	Yes	Yes	Yes
Cunningham et al.[22]	Yes	Yes	Yes	Yes	Can’t tell	Can’t tell	Yes	Yes	Yes	Yes
Lokin et al.[23]	Yes	Yes	Can’t tell	Yes	Yes	No	Can’t tell	No	Yes	Yes

### Patients’ experience of PAD

Using the chronic disease framework, the identified patients’ experiences described as themes, subthemes, categories, and patients’ quotes in included studies were mapped into experiences of diagnosis, experiences of undergoing interventions, experience of undergoing conservative management, post treatment experiences, and long term experiences. The themes, subthemes and categories, and the corresponding included studies that described them as presented in Table 3.

**Table 3:** Framework and themes from the synthesized experience of peripheral arterial disease.

	Galea et al. [10]	Harwood et al. [11]	Hallin et al. [12]	Schorr et al. [13]	Johnstone et al. [14]	Gibson & Kenrick et al.[15]	Egberg et al. [16]	Gorely et al. [17]	Treat-Jacobson et al. [18]	Wann-Hassan & Wennick et al. [19]	Wann-Hassan et al. [20]	Wann-Hassan et al. [21]	Cunningham et al. [22]	Lokin et al. [23]
**Experience of diagnosis**														
**Leg pain and cramp**		√	√	√	√		√	√	√		√			
**Fatigue**														
**Other discomforts**														
**Symptom dismissal**						√			√					
**Lack of knowledge**		√		√	√	√			√					
**Living in denial**					√	√	√	√	√					
**Delayed diagnosis**							√	√	√					
**Embarrassment**	√	√					√	√						
**Walking impairment**														
**Feeling restricted**														
**Loss of independence**			√	√	√	√	√	√			√			
**Impact of quality of life**		√	√	√		√	√		√		√			
**Sense of isolation & powerlessness**		√	√	√	√	√	√		√					
**Taking pain medication**				√		√	√	√			√			
**Avoiding/altering activity**				√		√	√	√			√			
**Treatment decision**	√					√								√
**Experience of undergoing surgical treatment**														
**Relived yet uncertain**								√		√			√	
**Lack of understanding/ knowledge**					√			√		√			√	
**Lack of information**					√			√		√			√	
**Fear of causing damage**													√	
**Patient experience of conservative treatment**														
**Walking not considered as therapy**	√	√						√					√	
**Varied outcome expectation**	√	√						√					√	
**Easily overcome by barrier to walking**	√	√						√					√	
**SEP was seen as friendly**		√												
**SEP provided structure**	√	√												
**Lack of knowledge**	√	√						√					√	
**Patients’ post treatment experience**														
**Temporary symptom relief**												√	√	√
**Disappointment over unmet expectation**												√	√	
**Lack of knowledge**										√			√	√
**Return of symptoms**						√						√	√	
**Long term experience**														
**Continued lack of understanding**														
**Adaptation to physical limitations**						√	√					√	√	
**Adjusting to deteriorating health**						√	√					√	√	
**Adjusting to deteriorating quality of life**									√		√	√	√	
**Life re-orientation**						√	√					√	√	

#### Patients’ experience of diagnosis

Patients’ experience of diagnosis summarises reports of patients’ experience of symptoms presentation, diagnostic processes, pre-treatment processes including consultations, and processes involved in making decisions about treatment. The emerging themes reflect patients’ feeling of leg pain, fatigue and other discomforts, which later became a burden, resulting in walking impairment, loss of independence and control. Patients described a common first experience of symptom of PAD as pain while walking usually beginning as a slow, creeping feeling of fatigue which gradually developed into pain and cramp[11–14,16–18,20]: “just like somebody gripping the calf muscle really tightly”, “crippling”, “stops me in my tracks”, and having a “wearing effect”[18]. Although the pain increased or became more frequent[15,16],patients initially dismissed symptoms as trivial[15,18], and sometimes thought it was a sign of old age[15,17]: “I never complained [to the doctor] because I didn’t think I had a complaint[15].

Symptom progression saw the pain and discomfort began to wear the patients down[11,15,16,18], often bringing embarrassment when they had to stop in the middle of the road to let the pain go away[10,11,16,17]. In addition to walking impairment, patients reported impairment in other aspects of physical functioning, feeling restricted and a loss of independence[12,14–17,20], which had a major impact on several aspects of their quality of life[11–13,15,18] including pleasure[16], social life[12,20], recreation[16], and sleep quality[20] for those with rest pain. Patients interpreted this as a sense of burden, powerlessness, isolation, and compromised independence: “Yes, it’s just like it has taken the strength and power from me…, you have to do this and that but you can’t … You have to give up”[20]. Although unsuccessfully, patients attempted to deal with their symptoms by taking pain medication, avoiding or altering their activity, taking slow steps or walking short distances[13,15–17,20]. Patients also demonstrated a limited understanding of their disease[11,14,17,18]: “I thought I had a muscle injury”[18].

There was substantial delay in diagnosing the condition, and getting treatment. The first component of the delay was patients initial dismissal of their symptoms for lack of knowledge about the disease[17–19], and consequent delay in seeking medical help[17,18]. Even when medical help was sought, many patients were frustrated at the time and effort required to gain definitive diagnosis[18]. “I ended up going to different doctors before I got to one that put his finger on the vascular end of it.…went through quite a little stuff for 3–4 years before I finally got the guy that said ‘You haven’t got any circulation”[18]. When eventually diagnosed, patients discussed receiving little attention and follow-up and had strong perception of lack of empathy or awareness of them as a person on the part of the healthcare professionals ‘‘When I actually got diagnosed, I was there 10 minutes, then it was this, this, this and that, right you’re discharged, that’s it, go”[17]. Patients desired specific information about their disease but reported a sense of being left alone with little specific information or advice. ‘‘I mean everything I’ve got I want to know about, and you don’t get to know, simple as that, you ask and you don’t get a full explanation”[17].

Generally, patients were passive in the treatment decision-making process and assumed that healthcare professionals to be competent, knowledgeable and acting in their best interest. “The likes of [surgeons], they know what they are talking about they don’t say things unless they are sure so I accept what they want to do”[15]. So when surgery was decided, patients had unrealistic expectations that surgery would solve all of their problems[15]. Walking was an overlooked first-line treatment strategy. Even when healthcare professionals had advised patients with IC to walk, patients did not consider it as a treatment. “There’s no treatment. I’m getting no treatment, not for this. I’m getting advice, and the advice is ‘try to walk through it’. That’s the only advice I’ve ever had”[10]. Similarly, regarding other lifestyle modification and risk factor management, although patients have been told about the risk of smoking, majority of patients did not understand the systemic nature of their disease or wider atherosclerotic risks. “I am a healthy person. I rarely suffer from something serious. No scary diseases and. no heart disease or whatever”[23].

#### Experience of undergoing surgical treatments

Patients’ experience of surgical acute care described surgery and other acute care interventions. Emerging themes reflected deficiency in the acute care orientation of surgical interventions for PAD. Patients discussed feeling relieved yet uncertain, and reported difficult understanding the technical language typically used by their surgeons[17,19,22]. Typical aspiration was for knowledge and information on self-management when returning home after surgical intervention but patients described receiving little, non-specific, or mixed information, often with no written material to take home[17,19,22], even when requested[19]. “It might be good to receive some of the information during the hospital stay because you have to wait quite a lot…”[19]. Not receiving specific information made patients confused and uncertain[19,22] and they interpreted it as lack of empathy[17].

Irrespective of the disease stage at the point of surgical intervention, patients described lack of information about their post-operative health and, for instance how to deal with the “nerve damages” and swelling[22]. In addition patients spoke about being discharged with insufficient knowledge about their disease and the importance of risk factor management. Hence patients lacked clear and specific understanding of how to effect important behaviour change (e.g. walking exercise) to reduce risks and/or improve symptoms[15,22]. Due to the short time of consultations and lack of clear information, patients reported not being able to get their questions answered by their health care professionals[19,22] but had resorted to other sources outside medical contact, including the internet, friends and family members, to gain knowledge about their condition[17,19]: “…and you don’t get to know, simple as that, you ask and you don’t get a full explanation”[17]. This uncertainty and lack of understanding led to many patients avoiding walking for fear of causing damage[22]

#### Patients experience of conservative treatments

Patient’s experience of conservative treatment relates to experience with walking treatments (and lifestyle modification), their perspective of walking treatment for PAD or walking behaviour after revascularisation. Emerging themes indicate many patients with IC do not consider walking exercise as important self-management opportunity either as first-line therapy or after undergoing revascularisation[10,11,17,22] This was either due to patients’ trivialisation of symptom[10] or feeling of powerlessness, frustration by the severity of their symptoms[10] or just lack of motivation to walk[17]. In addition lack of specific and tailored walking guidelines meant that patients had varied outcome expectations, and limited purposeful walking for exercise, and were easily overcome by barriers to walking to intensity[10,11,17,22]

Patients described experience of SEP for IC as “friendly,” “relaxing,” and one that gave them a “feeling of all being in it together”[11]. Completing SEP provided patients with structure and reassurance regarding safe and effective dose of walking[10], however, patients described comorbidities, leg pain, lack of motivation and lack of time, and the required commitment needed to attend 3x/week as barrier to participating in SEP[10,11,17]. In addition, they discussed that attending SEP did not aid them to walk independently in their community[10]. Patients however noted that symptom improvement following walking exercise encouraged them to go on[10]. Similar to acute surgical care treatments, a resonating theme among patients regarding walking was their desire for specific and tailored information, guidance and support to achieve the walking recommendations, and the lack of these in much of the walking advice received.

#### Patients’ post treatment experience

Patients’ post treatment experience intended to capture patients’ experiences in the short period (≤ 6 months) post-surgical interventions or conservative management, during which patients experienced rapid changes to their symptoms and learnt to navigate these changes. However, no study reported on patients’ experience of living with PAD post-conservative treatments. Emerging themes of post-surgery experience reflects the feeling of becoming better but not cured, gradual emergence of uncertainty, and fear, and then patients’ attempts to recapture control of their life.

Undergoing surgical interventions provided patients with temporary pain relief enabling them to walk unaided again, and to sleep well for patients with CLI[21]. With an unrealistic expectations that surgery had “cured everything”[23], patients focussed solely on this symptom relief, believing PAD to be acute and treatable, rather than a chronic condition which necessitates self-management[22]. However, the high hope of recovery progressed to disappointment over unmet expectations when symptoms remerged and/or other existing ailments began to unravel[21,22]. This highlights a continued lack of understanding of the aetiology and the systematic nature of PAD even when patients’ had undergone surgical interventions[19].

Patients discussed not receiving adequate information when returning home from surgery and were soon bothered with several questions. To them their major source of information and support is from consultation with their surgeons but this did not provide them with a good understanding of their disease[22]. They continued to be unclear about the systemic nature of their condition, and how to translate lifestyle change into managing their condition[23]: “But in some cases it would help if you could all calm down a little when you are talking to us. I have met doctors who say hmhmhm and you notice that they are stressed and then you have no further questions…”[19]. In the context where surgery was expected to be a complete cure, the return of claudication pain and cramps assumed greater significance[15,21,22] and patients were soon overtaken with feelings of uncertainty, and coming to an end point[21]: “It’s just the odd pain that frightens me… I say oh God I hope it’s not happening again”[15]. This marks a point where patients would be forced to accept the chronic nature of PAD.

#### Long term experience (Adapting to the “new normal”)

This captures the long-term experience of living with PAD, during which patients start adapting to disease limitation, and learning alternative strategies to maximise living including reappraising meaning in life. Although many patients have started coming to terms with the fact that they have a chronic condition, they continue to lack understanding of the disease in terms of the broader atherosclerotic risk. Patients were more bordered about their chance of going back to surgery or being offered one if they need it, than the risk of stroke and broader cardiovascular events[22]. The majority have accepted that they will always be dependent on help and support, but felt frustrated and helpless at having to watch their role being taken over by spouses and children[21].

Patients discussed their resolve to begin adaptation to their physical limitation, and adjusting their outlook within their limited activity and deteriorating health[15,16,21,22]. “I don’t go down, sit in a corner, and cry every day. That wouldn’t make it any more fun”[21]. Often a complex process, patients expressed mixed feelings and varied ability to make this adaptation[18], often requiring them to reorient life, and accept with resignation the fact of giving up some important things including hobbies, leisure, and social life[20,22]. For many, there was a feeling of being forced to reappraise the meaning of life and to develop a new value in order to make the best of their situation. “But to fully accept it is hard, you can put it out of your mind for a while but then the thoughts come again, why you can’t walk properly”[21].

Patients began to learn how to live with the remaining circulation problem in the leg and are more moderate about their expectation[21]. “Yes, I suppose you think less and less about it and realise that you’re like this and it doesn’t seem like it’s going to work out much better”[21]. To remain fairly active within the restriction placed by PAD, patients had begun to walk more slowly and to sit down instead of standing for gardening[21].

## Discussion

This paper presents a systematic review and framework synthesis of qualitative data of patients’ experiences of PAD. Findings highlight the considerable impact of living with PAD on patients, and the need for practice to incorporate these constructs into care pathways. Most of the studies reported on experiences of patients waiting for, undergoing, or following vascular surgery/intervention with little attention given to patients who were undergoing conservative management. Practice recommends conservative management (including supervised or home based exercise, medical therapies and lifestyle modification) either as first line treatment or as adjunct for people with PAD[28]. Thus, there is a need for research to understand the experiences of this population who are undergoing conservative management as these may differ from those who are undergoing surgery.

Altered sensation, typically presenting as pain in the leg while walking, was a common first concern[15–18]. Although evidence indicate that many patients with PAD including some with moderately severe disease report no, or atypical symptoms[29,30], the review supports several articles reporting common symptoms of PAD[31,32].

Patient experience of substantial delay in diagnosis and treatment, and the difficulty in navigating health care information reflects current PAD literature[33]. The fact that patients delayed seeking medical help until there was significant walking impairment and restrictions in many aspects of their life[16,17], could be indicative of a lack of understanding of symptom/disease pathology in this population[34]. To reduce the risk of complications such as critical limb ischaemia, the need for limb amputation or adverse events (cardiovascular disease morbidity/mortality) prompt diagnosis of PAD is critical[35]. As seen in the included studies[10,18,23], and elsewhere in the literature[34,35], lack of understanding of the disease pathology is common throughout PAD disease trajectory even among patients who have undergone intervention or SEP. In addition to aggressively pursuing the NICE recommendation to provide information on the cause, severity, associated risks, risk factors, pain management, treatment options, and psychosocial support for persons already diagnosed with PAD[36], a wider campaign may help raise general awareness of PAD and atherosclerosis to improve recognition of early symptoms. Despite being an independent risk factor for stroke, heart failure hospitalization, and other cardiovascular events and deaths[37], and having huge impact on patients quality of life[38], studies in several countries indicate that population level knowledge about PAD, is very low[34,39–41]. Similarly, given that typical PAD symptoms may not be always present, or when present may not be reported early by patients, routine screening of persons with risk factors is important, and may reduce the delay in diagnosis and treatment[42].

Central to the findings related to patients’ encounter with acute care treatments is the desire for clear, consistent, and specific information about their condition, and the failure of health care professionals in meeting this requirement. Patients felt uninvolved in the decisions about treatment, and usually had incorrect or unrealistic expectations of the planned intervention. The need for patients with PAD to participate in decisions regarding treatment has been highlighted[43]. It appears there is a missed opportunity during patients’ interaction with healthcare providers. Patients do not gain the information that would not only enable them make decision about choice of care but also to cultivate important lifestyle behaviours to affect risk reduction and symptom improvement. One way of making use of this opportunity might be a well-designed structured education programme made available for patients irrespective of their choice of treatment. This has been implemented in diabetes[44] and has been advocated for adoption for patients with PAD[45].

Post-treatments challenges faced by participants reflected the inherent weakness of the cure approach to management of PAD. Patients’ excitement over symptom relief, including improved walking ability, usually faded away gradually with the disappointment of the return of pain and the unmet expectation of the ability of surgery to cure everything. For patients who have undergone SEP, the realisation that they could not replicate the gained treadmill walking ability in an independent improved walking behaviour in the community was a major discouragement to incorporate walking exercise into their daily life. Indeed patients were still lacking self-management strategies, and the knowledge and skill to navigate the new reality of their condition, and soon began to struggle at this awareness of PAD as a chronic condition[21,22]. Many were afraid uncertain about what is safe and beneficial within the walk-limiting pain and multiple commodities. The role of walking limiting pain [46–48], comorbid health concerns[46,47,49–53], and lack of knowledge[47,54] as barriers to adopting self-management strategy in patients with symptomatic PAD are well documented[55]. Instead of a hurried discharge from the surgery or at the end of SEP, it may be more beneficial to arrange a patient education program, perhaps in an outpatient setting, to teach or improve patients’ self-management skills as part of an overall long-term secondary prevention approach similar to that in other long-term, progressive conditions.

The long-term experience of living with PAD sees patients begin to find ways to adapt to the disease limitations. This transition meant that patients had to deal with shrinking physical, social and psychological horizons due to their physical limitation[15], through a process of downward comparison that enabled them see the positive side of their situation[21]. Downward comparisons refer to when a patient compares to another who is perceived to be ‘worse off’[56]. Chronically ill patients often compare with each other during group interactions potentially providing an opportunity for them to modify their self-perceptions and adjust to ongoing threats to their health including psychological and physical well-being[56]. Patients’ capacity to use downward comparison in coping with the transition could be explored during self-management programmes in PAD.

Our findings highlight the paucity of research reporting on patients’ experience of undergoing conservative treatments. Particularly, no qualitative study reported on account specific to experiences of undergoing conservative medical treatments or long-term experience following SEP. Although one study[23] of patients who have undergone either SEP or PTA found that patients disease understanding remained poor after 6–18 months, it was not clear about account specific to which group. Given that conservative treatments including medical managements and SEP are central to PAD disease management pathway, further research should focus on how patients navigate these care options.

### Limitations

There are several limitations to this review. Only English language articles were included, with most of the included studies carried out in Europe, putting limitations to the understanding of a more global picture of the experiences of living with PAD. Secondly, the issue of participants’ relationship with the researcher need to be adequately considered and clarified in future studies of experiences of PAD. Lastly, many included studies reported data on patients across the breadth of experiences and varied stages of the disease. It is acknowledged some constructs may be subtle and saliently unique to each stage of disease or treatment type, hence attempts were made, where possible, to map reported findings to each stage of diseases and management indications. Given that many included studies reported heterogeneous patient sample at different stages of the disease or undergoing different treatments, it is appreciated that this preliminary framework may have missed important subtleties unique to certain treatments or certain disease stage. However, many themes identified in the review were common throughout the illness trajectory and may be relevant in addressing management of PAD irrespective of stage/treatment. This review is considered novel, pragmatic, and useful for the purpose of unpacking experiences unique to each stage, or extent of illness, and will be useful in planning care in this patient population irrespective of disease stage or treatment.

### Conclusion

The findings contribute to a greater understanding of the experience of living with PAD from the patients’ perspectives and highlights general lack of disease understanding across the disease trajectory, the delay in getting diagnosis and treatment, the physical limitation, social and psychological burden and demand on the patients. Although certain experiences were common throughout the illness trajectory, findings indicate that some might be unique to a particular stage or following certain treatments. These findings have implication for designing interventions for people with PAD as well as in selecting comprehensive patient reported outcomes for intervention in this population. In addition, there is a paucity of literature concerning experience of patients with PAD who are undergoing conservative treatments. Given that conservative management is a key recommended in PAD disease, future research is needed to better understand how patients engage with conservative management.

## Supporting information

S1 Table PRISMA 2009 checklist patients experiences of living with peripheral arterial disease. (DOC)

S1 Fig A search strategy to identify articles of patient experience of living with peripheral arterial disease implemented in web of science. (DOCX)
