# Supplementary material for: Living a burdensome and demanding life: A qualitative systematic review of the patients experiences of peripheral arterial disease
Source: PLoS One. 2018 Nov 15;13(11):e0207456. doi: 10.1371/journal.pone.0207456 (PMC6237376; doi:10.1371/journal.pone.0207456)
Supplement: S1 Fig — (DOCX) [file pone.0207456.s002.docx]

| 1. (Intermittent claudication* or peripheral arterial occlusion* or peripheral arterial disease* or peripheral vascular disease* carotid artery stenosis* or aortic aneurysm abdominal* or lower limb ischaemia) 2. (quality of life* or life experience* or lived experience* or patient reported experience* or patient experience* or living experience* or illness beliefs* or llife satisfaction* or symptom experience*) 3. (qualitative research* or qualitative studies* or mixed method studies* or peripheral vascular disease experience* or experience of walking with peripheral arterial disease*) 4. #1 AND #2 AND #3 (Qualitative studies) 5. #1 AND #2 AND #3 (mixed method studies) |
| --- |

Search updated 15/02/2018

S1 Fig. A search strategy to identify articles of patient experience of living with peripheral arterial disease implemented in web of science
